# Transient Platelet Satellitism and Cellular Phagocytosis Associated With Type A Influenza Virus Infection: A Case Report

**DOI:** 10.7759/cureus.79882

**Published:** 2025-03-01

**Authors:** Panagiota Tsiatsiou, Vasiliki Tsaireli, Michail Makris, Georgia Kaiafa, Lemonia Skoura

**Affiliations:** 1 Laboratory of Medicine, Department of Microbiology, American Hellenic Educational Progressive Association (AHEPA) University Hospital of Thessaloniki, Aristotle University of Thessaloniki, Thessaloniki, GRC; 2 First Propedeutic Department of Internal Medicine, American Hellenic Educational Progressive Association (AHEPA) University Hospital of Thessaloniki, Aristotle University of Thessaloniki, Thessaloniki, GRC; 3 Laboratory of Hematology, Department of Microbiology, American Hellenic Educational Progressive Association (AHEPA) University Hospital of Thessaloniki, Aristotle University of Thessaloniki, Thessaloniki, GRC

**Keywords:** peripheral blood smear, phagocytosis, platelet satellitism, thrombocytopenia, type a influenza infection

## Abstract

Despite advancements in fifth-generation hematological analyzers, the microscopic examination of peripheral blood smears remains crucial for diagnosing thrombocytopenia. This study aims to report a rare case of transient platelet satellitism and cellular phagocytosis while emphasizing their clinical implications, particularly in the misdiagnosis of thrombocytopenia during viral infections. We present the case of a man with type A influenza infection-associated pneumonia and thrombocytopenia, in whom platelet satellitism formation and cellular phagocytosis were observed on a blood smear. Following appropriate diagnostic procedures, the platelet count was corrected, thereby preventing unnecessary therapeutic interventions and emphasizing the significance of accurate diagnostic techniques to avoid overtreatment. The simultaneous presence of platelet satellitism and cellular phagocytosis resolved after therapeutic measures. This case underscores the critical role of peripheral blood smear examination in the diagnostic process and calls for further research into the mechanisms behind platelet satellitism and cellular phagocytosis in viral infections. Accurate diagnosis is essential, particularly in distinguishing pseudothrombocytopenia, an in vitro artifact, from genuine medical conditions.

## Introduction

Platelet satellitism is an uncommon in vitro phenomenon observed in peripheral blood smears prepared from ethylenediaminetetraacetic acid (EDTA)-anticoagulated samples [1]. It is not associated with any specific disease process, and the underlying mechanisms remain incompletely understood, though they likely involve immunological factors such as autoantibodies [2,3]. It is hypothesized that autoantibodies, typically triggered by EDTA exposure, bind to hidden antigens on the platelet surface. This binding promotes platelet clustering around neutrophils via interactions with the leukocyte Fcγ receptor III (CD16) [4]. The incidence of this EDTA-related phenomenon is estimated to range from 0.03% to 0.27% in the general population [5]. The process is characterized by platelet adhesion to polymorphonuclear leukocytes, culminating in a distinctive configuration that resembles a rosette-like structure [6,7]. The aggregation of platelets induced by EDTA occurs because EDTA chelates divalent cations essential for platelet stability, which alters the conformation of platelet membrane glycoproteins, particularly glycoprotein IIb/IIIa. This alteration triggers immunoglobulin-mediated reactions, leading to platelet clumping. Notably, this phenomenon is absent in blood specimens collected using alternative anticoagulants, such as heparin, citric acid, dextrose oxycitrate, or ammonium oxalate. Therefore, platelet satellitism formation is directly linked to the specific anticoagulant employed during blood collection. Cellular phagocytosis is another distinct biological process particularly prevalent in immune cells, such as macrophages and neutrophils, which are vital components of the innate immune system. The phagosome, a specialized vesicle within phagocytes, macrophages, or neutrophils, plays a crucial role in the phagocytic process, enabling the immune cells to identify, adhere to, and internalize target entities [8,9]. A cell can engulf or ingest both cells of its own type and those of different types. This critical process of the immune system serves various functions, including defense, nutrient acquisition, and the elimination of cellular debris [10-13].

## Case presentation

**Figure 1:**
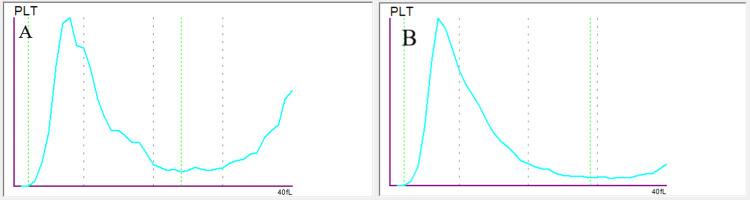
Histogram of platelets on (A) presentation (EDTA) and (B) the fifth day of therapy (EDTA). EDTA: ethylenediaminetetraacetic acid.

A 79-year-old man presented with respiratory symptoms, including cough, fever, and dyspnea, which began the day before admission. Chest X-ray revealed pneumonia, and molecular testing via polymerase chain reaction (PCR) (Xpert® Xpress CoV-2/Flu/RSV (respiratory syncytial virus) plus system, Cepheid, Sunnyvale, CA, United States) confirmed the presence of influenza A virus. During hospitalization, routine hematological analysis revealed thrombocytopenia. The patient's medical history, as reported by his family doctor, was unremarkable, without any autoimmune disorder, bleeding tendency, or prior instances of thrombocytopenia. A blood sample was collected using EDTA as an anticoagulant. Laboratory tests showed a hemoglobin level of 11.8 g/dL, a white blood cell count within the reference range with a predominance of polymorphonuclear cells, and a platelet count of 75,000/μL (XN-10, Sysmex Corporation, Kobe, Hyogo, Japan). The C-reactive protein (CRP) level was 22.8 mg/dL (normal value, <0.5 mg/dL). The platelet histogram displayed a serrated ("saw-teeth") curve with the largest platelet aggregates (Figure 1). Platelet flags triggered alerts for thrombocytopenia and platelet clumping. Microscopic examination of the peripheral blood smear after May-Grunwald Giemsa staining revealed platelet satellitism around most neutrophils and lymphocytes, and occasionally monocytes, along with cellular phagocytosis. To further investigate, we repeated the complete blood count using alternative anticoagulants, such as sodium citrate and heparin, and prepared two direct samples without anticoagulants.

A comparative study of platelet satellitism was conducted using the following samples: (a) blood with EDTA as an anticoagulant, (b) blood with sodium citrate as an anticoagulant, (c) blood with heparin as an anticoagulant, and (d) direct smears without anticoagulant (Table 1). The patient received a five-day course of oseltamivir carboxylate therapy. As his condition improved, laboratory parameters also normalized, and he was discharged on the seventh day. Following the therapy, the same procedure with different anticoagulants was repeated (Table 2).

**Table 1:** Complete blood count on presentation.

	On presentation: sample with EDTA	On presentation: sample with sodium citrate	On presentation: sample with heparin	Units	Normal range
White blood cells	10.33	7.66	7.89	K/μL	3.8-10.5
Neutrophils	8.7	6.05	6.15	K/μL	1.6-6.5
Lymphocytes	1.28	1.27	1.38	K/μL	1.5-3.6
Monocytes	0.24	0.31	0.45	K/μL	0.2-1
Eosinophil	0.00	0.00	0.00	K/μL	0.0-0.7
Basophil	0.004	0.03	0.04	K/μL	0.0-0.2
Red blood cells	4.14	4.69	4.52	M/μL	4.2-6.3
Hemoglobin	11.8	13.3	12.9	g/dL	14-18
Hematocrit	34.9	40.7	38.8	%	40-52
Platelets	75	170	185	K/μL	150-450
Fluorescence platelet count (PLT-F)	124	176	175	K/μL	150-450
Mean platelet volume (MPV)	11.5	10.8	10.8	fL	7.5-11
Platelet distribution width (PDW)	13.0	13.0	13.0	fL	12-17.5
Plateletcrit (PCT)	0.090	0.21	0.022	%	0.15-0.35
Platelet-to-large cell ratio (p-LCR%)	34.8	30.4	31.5	%	13-43
PLT/flag	Thrombopenia/platelet clumping	None	None		

**Table 2:** Complete blood count on the fifth day of therapy.

	On the fifth day of therapy: sample with EDTA	On the fifth day of therapy: sample with citrate	On the fifth day of therapy: sample with heparin	Units	Normal range
White blood cells	8.85	8.25	8.65	K/μL	3.8-10.5
Neutrophils	5.5	5.3	5.5	K/μL	1.6-6.5
Lymphocytes	2.70	2.08	2.15	K/μL	1.5-3.6
Monocytes	0.41	0.57	0.63	K/μL	0.2-1
Eosinophils	0.19	0.29	0.35	K/μL	0.0-0.7
Basophils	0.04	0.05	0.06	K/μL	0.0-0.2
Red blood cells	4.02	3.28	3.52	M/μL	4.2-6.3
Hemoglobin	11.5	9.6	10.1	g/dL	14-18
Hematocrit	33.1	28.4	28.9	%	40-52
Platelets	240	247	251	K/μL	150-450
Fluorescence platelet count (PLT-F)	245	258	257	K/μL	150-450
Mean platelet volume (MPV)	12.0	10.8	10.8	fL	7.5-11
Platelet distribution width (PDW)	15.0	12.0	12.0	fL	12-17.5
Plateletcrit (PC)T	0.27	0.270	0.27	%	0.15-0.35
Platelet-to-large cell ratio (p-LCR%)	29.6	30.8	31.2	%	13-43
PLT/flag	None	None	None		

**Figure 2:**
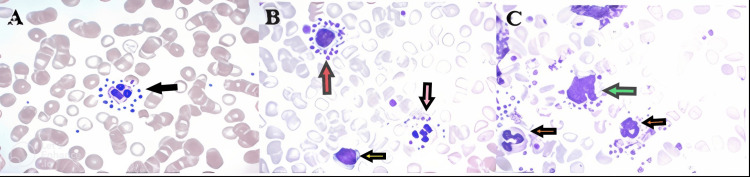
Microscope images with platelet satellitism and phagocytosis (×1000 magnification). (A) Polymorphonuclear neutrophil (black arrow). (B) Lymphocyte (red arrow) and a polymorphonuclear neutrophil (pink arrow) with satellitism and a lymphocyte (yellow arrow) without satellitism. (C) Monocyte (green arrow) and two polymorphonuclear neutrophils (orange arrows).

**Figure 3:**
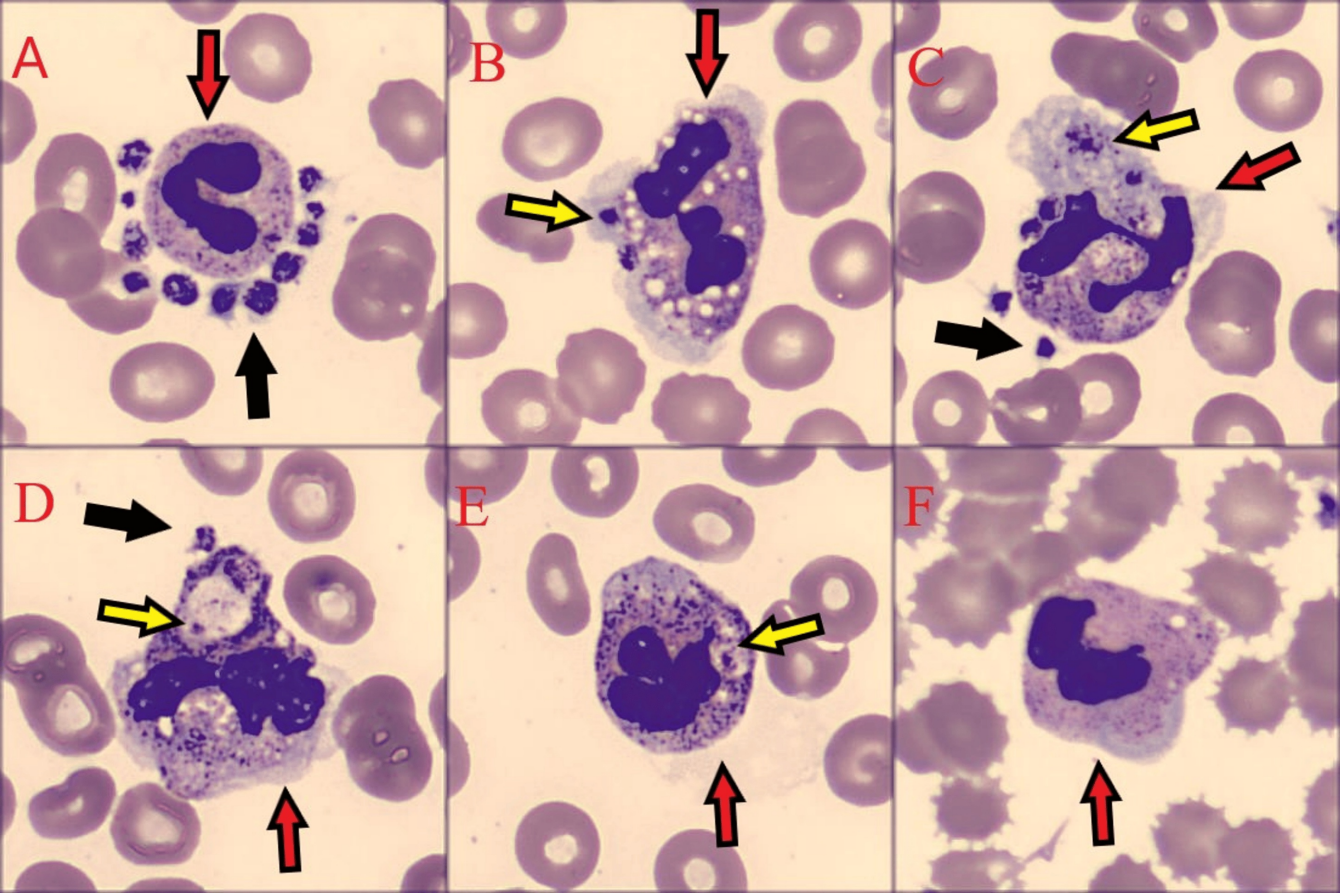
Images from DI-60 (CellaVision, Lund, Sweden). (A-E) Platelet satellitism. (F) Cellular phagocytosis. Image with citrate on presentation. Red arrows indicate polymorphonuclear neutrophils, black arrows indicate platelets, and yellow arrows indicate platelet phagocytosis from polymorphonuclear neutrophils.

Our findings confirm the transient nature of platelet satellitism in EDTA-anticoagulated blood samples, specifically during the course of the infection. The examination identified the cell types involved (mainly neutrophils, with occasional lymphocytes and monocytes), as well as phagosomes containing platelets within some neutrophils (Figure 2 and Figure 3). After completing the treatment course, the patient showed improvements in both clinical presentation and platelet count. Complete blood counts with three alternative anticoagulants (EDTA, citrate, and heparin) yielded consistent results. The platelet histogram exhibited a narrow distribution with a distinct peak, returning to baseline at 20 fL (Figure 1). Furthermore, no flags of thrombocytopenia or platelet clumping were detected. Subsequent microscopic analysis of the blood smear revealed no evidence of platelet satellitism around the leukocytes. Additionally, CRP levels normalized to within reference ranges.

## Discussion

This case report highlights the importance of the peripheral blood smear as a crucial diagnostic tool for evaluating thrombocytopenia. By examining platelet morphology, clustering, or associations with white blood cells, conditions such as pseudothrombocytopenia due to platelet clumping can be excluded. The absence of schistocytes helps rule out thrombotic microangiopathies such as thrombotic thrombocytopenic purpura (TTP), hemolytic uremic syndrome (HUS), or disseminated intravascular coagulopathy (DIC). Similarly, the absence of blasts or nucleated red blood cells may exclude bone marrow disorders like leukemia, while giant platelets and white cell inclusions rule out hereditary thrombocytopenias. Furthermore, immune thrombocytopenia, a diagnosis of exclusion, can be ruled out by the presence of platelet satellitism. Though rare, platelet satellitism can lead to an incorrect diagnosis of thrombocytopenia (pseudothrombocytopenia), particularly when evaluations rely solely on automated hematology analyzers without simultaneous microscopic examination of the peripheral blood smear. A limited number of documented cases involving transient thrombocytopenia, platelet satellitism formation, and phagocytosis, particularly in the context of viral infections, have been described in the literature. Understanding this phenomenon and its transient nature is paramount for the accurate interpretation of test results. Automated hematology analyzers may report artificially diminished platelet counts due to this condition. Misinterpretation of hematology analyzer outputs, including warning flags and histograms, combined with the lack of routine blood smear examination, often leads to misdiagnosis. As a result, this condition frequently goes unrecognized in clinical settings. Furthermore, in viral infections, such as type A influenza in our case, further research is necessary to understand the underlying processes of platelet satellitism formation and cellular phagocytosis. Such investigations are essential for determining the clinical relevance and potential consequences in analogous cases.

## Conclusions

This study emphasizes the importance of laboratory staff being aware of and following appropriate diagnostic protocols. Furthermore, it is essential to educate clinicians about the potential of this laboratory artifact to prevent unnecessary medical interventions. Effective communication between laboratory staff and healthcare providers is also crucial for discussing unexpected or atypical test results, which can improve patient care and optimize resource utilization.
